# Fisher Information Density Functional Theory

**DOI:** 10.1002/jcc.70215

**Published:** 2025-08-25

**Authors:** Á. Nagy

**Affiliations:** ^1^ Department of Theoretical Physics University of Debrecen Debrecen Hungary

**Keywords:** density functional theory, Fisher information density, variational principle

## Abstract

According to the density functional theory, the density contains sufficient information to compute the value of any observable. It is shown that the Fisher information density also includes this knowledge. The Fisher information density functional theory is constructed. The variational principle is extended to the energy as a functional of the Fisher information density. Hohenberg‐Kohn‐like theorems are shown to be valid.

## Graphical abstract

According to the density functional theory the density contains all information about the system. It is now revealed that the Fisher information density also includes all the knowledge of the system. The variational principle stands for the Fisher information density, too. Therefore, the Fisher information density functional theory can be established. Hohenberg‐ Kohn‐like theorems are derived. As an illustration, the Fisher information density and the density of the harmonic two‐electron molecule are presented.

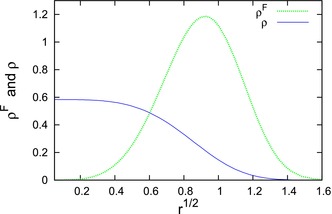

## 1 Introduction

Density functional theory (DFT) has benefited a lot from information‐theoretical concepts (see, e.g., [1, 2, 3, 4, 5, 6, 7, 8, 9, 10, 11, 12, 13, 14, 15, 16]). As the electron density can be considered proportional to a probability distribution function, information quantities such as Shannon [17] and Rényi [18] entropies or Fisher information [19] can be determined from the electron density. The relationship between the information‐theoretical approach and DFT allowed significant conceptual and practical development. It led to a better understanding among others, reactivity, and chemical bonding. For example, the Euler equation of DFT can be derived from the principle of extreme physical information, that is, applying the variational principle to the Fisher information with certain conditions [20, 21]. Liu and coworkers initiated information functional theory [22] and demonstrated that several electronic properties can be accurately described by information‐theoretic quantities.

Besides the information quantities, certain information densities have also been studied. Moreover, it has been shown that the Shannon entropy density can be used as a descriptor of a Coulomb system [23]. It means that, in the knowledge of the Shannon entropy density, we can determine the Hamiltonian, and after solving the Schrödinger equation, any property of the system can be computed.

In this paper, it is shown that the Fisher information density defined by Equation (5) below is also a descriptor of a Coulomb system. Moreover, the density can be directly determined if the Fisher information density is known—that is, without solving the Schrödinger equation. If the system is spherically symmetric, the density can be computed by an integral containing the Fisher information density (Equation (22) below). If there is no spherical symmetry, the relationship between the density and the Fisher information density is more sophisticated: The square root of the density and the square root of the Fisher information density satisfy the so‐called eikonal equation. In addition, the DFT variation principle also applies to the Fisher information density. So, we can conclude that the Fisher information density contains all information on the system.

The paper is arranged as follows: Section 2 contains the definition of the Fisher information and the Fisher information density. In Section 3, it is shown that the Fisher information density is a descriptor of a Coulomb system. Spherically symmetric systems with local external potential are studied in Section 4. The general case is presented in Section 5. The variational principle is extended to the energy as a functional of the Fisher information density in Section 6. Sections 7 and 8 present the Discussion and the Conclusion.

## 2 Fisher Information

Fisher information [19] for the probability distribution function f(r) is defined as

(1) $$\begin{array}{l} I^{F}=\int \frac{|\nabla f(\text{r}){|}^{2}}{f(\text{r})}d\text{r} \end{array}$$

(For more details, see References [20, 24].) For the electron density ϱ(r)

(2) $$\begin{array}{l} I^{F}=\int \frac{|\nabla \varrho (\text{r}){|}^{2}}{\varrho (\text{r})}d\text{r} \end{array}$$

is a measure of the spatial change of the electron density. $I^{F}$ is proportional to the Weizsäcker kinetic energy [25]:

(3) $$\begin{array}{l} T_{w}=\frac{1}{8}I \end{array}$$

an important ingredient of the kinetic energy functional. $T_{w}$ proved to be valuable in chemistry: It gives the so‐called steric term [26]

(4) $$\begin{array}{l} E_{steric}=T_{w} \end{array}$$

signifying that an atom occupies a certain amount of space in a molecule.

We define the Fisher information density (or local information density) [24] as

(5) $$\begin{array}{l} \varrho^{F}(\text{r})=\frac{|\nabla \varrho (\text{r}){|}^{2}}{\varrho (\text{r})} \end{array}$$

As it is shown in the following sections, it is not only a natural but an adequate definition, because a Fisher information density functional theory can be built on it.

## 3 Fisher Information Density as a Descriptor of Coulomb Systems

Consider first the Coulomb external potential. The Hamiltonian can be written as

(6) $$\begin{array}{l} {Ĥ}^{Coul}=\hat{T}+{\hat{V}}_{ee}+\overset{N}{\underset{i=1}{\sum }}v^{Coul}({\text{r}}_{i}) \end{array}$$

where $\hat{T}$ and ${\hat{V}}_{ee}$ are the kinetic energy and the electron‐electron energy operators. The Coulomb external potential has the form

(7) $$\begin{array}{l} v^{Coul}(\text{r})=\overset{M}{\underset{\alpha =1}{\sum }}v_{\alpha }^{Coul}(\text{r}) \end{array}$$

where

(8) $$\begin{array}{l} v_{\alpha }^{Coul}(\text{r})=-\frac{Z_{\alpha }}{\left|\text{r}-{\text{R}}_{\alpha }\right|} \end{array}$$

$R_{\alpha }$ and $Z_{\alpha }$ are the position vectors and the atomic numbers of the nuclei. N and M denote the number of electrons and nuclei.

Theorem 1 *The Fisher information density satisfies Kato's theorem* [27, 28, 29]

(9) $$\begin{array}{l} \frac{1}{\varrho^{F}(\text{r}={\text{R}}_{\alpha })}{\left.\frac{\partial \varrho^{F}}{\partial |\text{r}-{\text{R}}_{\alpha }|}\right|}_{\text{r}={\text{R}}_{\alpha }}=-2Z_{\alpha } \end{array}$$

Proof of Theorem 1 As it was shown (e.g., [30]), the leading term in Equation (7) is given by $v_{\alpha }^{Coul}$ Equation (8) if $\text{r}\to {\text{R}}_{\alpha }$, so the density is hydrogen‐like at the close vicinity of the nucleus α:

(10) $$\begin{array}{l} \varrho \propto e^{-2Z_{\alpha }|\text{r}-{\text{R}}_{\alpha }|} \end{array}$$

Therefore, the Fisher information density is also hydrogen‐like

(11) $$\begin{array}{l} \varrho^{F}=4Z_{\alpha }^{2}\varrho \end{array}$$

in the neighborhood of the nucleus α. Kato's theorem for the density has the form

(12) $$\begin{array}{l} \frac{1}{\varrho (\text{r}={\text{R}}_{\alpha })}{\left.\frac{\partial \varrho }{\partial |\text{r}-{\text{R}}_{\alpha }|}\right|}_{\text{r}={\text{R}}_{\alpha }}=-2Z_{\alpha } \end{array}$$

Therefore, $\varrho_{F}$ satisfies (9).

Theorem 2 *The Fisher information density determines the electron number* N.

Proof of Theorem 2 We follow the argument of Ayers on the shape function [31]. It is well‐known that the asymptotic behavior of the density is [32, 33, 34, 35, 36, 37, 38]

(13) $$\begin{array}{l} \varrho \propto r^{2b}e^{-cr} \end{array}$$

where

(14) $$\begin{array}{l} b=\frac{Z_{tot}-N+1}{\sqrt{2I}}-1 \end{array}$$

(15) $$\begin{array}{l} c=\sqrt{8I} \end{array}$$

and

(16) $$\begin{array}{l} I=E_{0}^{N-1}-E \end{array}$$

is the ionization potential. $E_{0}^{N-1}$ is the ground‐state energy of the N−1 electron system.

(17) $$\begin{array}{l} Z_{tot}=\underset{\alpha }{\sum }Z_{\alpha } \end{array}$$

is the sum of the atomic numbers. From Equations (5) and (13) we arrive to the asymptotic form of the Fisher information density:

(18) $$\begin{array}{l} \varrho^{F}\propto r^{2b}\left(\frac{2b}{r}-c\right)e^{-cr} \end{array}$$

Using Equations (14), (15), and (18) we are led to

(19) $$\begin{array}{c} \begin{array}{l} N=1+Z_{tot}+\frac{1}{4}\underset{r\to \infty }{\lim}\left\{\frac{\partial \left(\ln\varrho^{F}\right)}{\partial r}\left(2+\frac{\partial \left(\frac{\partial ln\varrho^{F}}{\partial r}\right)}{\partial \left(\frac{1}{r}\right)}\right)\right\} \end{array} \end{array}$$

That is, $\varrho^{F}$ determines N.

Consequently, the first Hohenberg‐Kohn theorem is valid for the Fisher information density.

## 4 Spherically Symmetric Systems With Non‐Coulombic Local External Potential

Turn now to non‐Coulombic local external potentials and consider the first spherically symmetric case. If the density is spherically symmetric Equation (5) takes the form

(20) $$\begin{array}{l} \varrho^{F}(r)=\frac{1}{\varrho (r)}{\left(\frac{d\varrho (r)}{dr}\right)}^{2} \end{array}$$

Equation (20) can be rewritten as

(21) $$\begin{array}{l} \varrho^{F}(r)=4{\left(\frac{d{\left[\varrho (r)\right]}^{1/2}}{dr}\right)}^{2} \end{array}$$

If the Fisher information density $\varrho^{F}$ is known, the density ϱ can easily be determined from Equation (21):

(22) $$\begin{array}{l} \varrho (r)=\frac{1}{4}{\left[\int_{\infty }^{r}(\varrho^{F}{\left((r^{\prime })\right)}^{1/2}dr^{\prime }\right]}^{2} \end{array}$$

The density determines the external potential; therefore, the Fisher information density also contains this information. Consequently, the first Hohenberg‐Kohn theorem is valid for the Fisher information density.

The simplest example is the H‐like ions with atomic number Z. One can easily check that the density $\varrho =\frac{Z^{3}}{\pi }e^{-2Zr}$ and the Fisher information density $\varrho^{F}=4Z^{2}\varrho =4\frac{Z^{5}}{\pi }e^{-2Zr}$ satisfy Equation (22).

Another analytical example is the harmonic two‐electron molecule [39], the analogue of the hydrogen molecule. In this model, we have a harmonic external potential and harmonic interaction between the two particles. The Hamiltonian is

(23) $$\begin{array}{l} Ĥ=-\frac{1}{2}\overset{2}{\underset{i=1}{\sum }}\nabla_{i}^{2}+\frac{1}{2}b\overset{2}{\underset{i=1}{\sum }}\left[{\left({\text{r}}_{i}-\frac{\text{d}}{2}\right)}^{2}+{\left({\text{r}}_{i}+\frac{\text{d}}{2}\right)}^{2}\right]+\frac{1}{2}\kappa {\text{r}}_{12}^{2} \end{array}$$

where d is the distance between the two centers, b is the harmonic force constant and ${\text{r}}_{12}={\text{r}}_{1}-{\text{r}}_{2}$. This model can also be called “Moshinsky molecule”. Ĥ can be reshaped as

(24) $$\begin{array}{l} Ĥ=\overset{2}{\underset{i=1}{\sum }}\left[-\frac{1}{2}\nabla_{i}^{2}+\frac{1}{2}k{\text{r}}_{i}^{2}\right]+\frac{1}{2}\kappa r_{12}^{2}+\frac{1}{4}kd^{2} \end{array}$$

where k=2b. Equation (24) is the Hamiltonian of the “Moshinsky atom” (e.g., [40, 41, 42, 43, 44]) aside from the constant $\frac{1}{4}kd^{2}$. That is, the “Moshinsky molecule” is almost the same as the “Moshinsky atom”. The ground‐state density is [43]

(25) $$\begin{array}{l} \varrho_{0}=2{\left(\frac{\tilde{\omega }}{\pi }\right)}^{3/2}e^{-\tilde{\omega }r^{2}} \end{array}$$

where

(26) $$\begin{array}{l} \tilde{\omega }=4\frac{\omega \omega_{0}}{\omega +\omega_{0}} \end{array}$$

(27) $$\begin{array}{l} \omega_{0}=\frac{1}{2}\sqrt{k} \end{array}$$

and

(28) $$\begin{array}{l} \omega =\sqrt{\omega_{0}^{2}+\kappa /2} \end{array}$$

That is, interestingly, the density of this harmonic two‐electron molecule is spherically symmetric. The density (25) is normalized to 2 as in DFT ϱ integrates to the number of particles. From Equations (20) and (25), we obtain the Fisher information density

(29) $$\begin{array}{l} \varrho^{F}={\left(2\tilde{\omega }r\right)}^{2}\varrho =2{\left(2\tilde{\omega }r\right)}^{2}{\left(\frac{\tilde{\omega }}{\pi }\right)}^{3/2}e^{-\tilde{\omega }r^{2}} \end{array}$$

**FIGURE 1:**
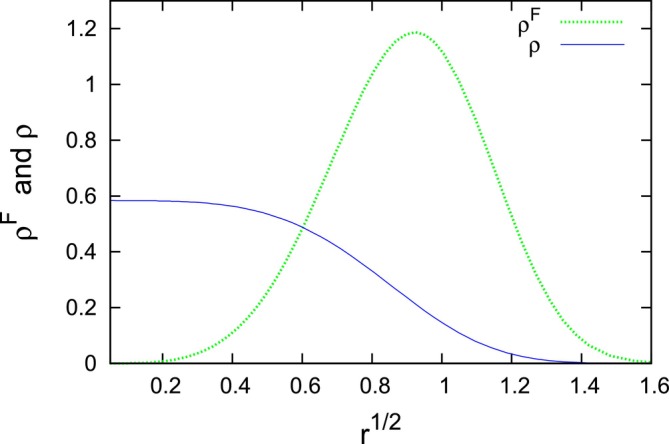
(Color online) The Fisher information density and the density of the harmonic two‐electron molecule (κ/k=2).

Obviously, Equations (25) and (29) satisfy Equation (22). Figure 1. presents $\varrho^{F}$ and ϱ for κ/k=2.

Observe that if r=0, we are led to an expression for the density at the nucleus expressed with the integral of the Fisher information density:

(30) $$\begin{array}{l} \varrho (r=0)=\frac{1}{4}{\left[\int_{\infty }^{0}(\varrho^{F}{\left((r^{\prime })\right)}^{1/2}dr^{\prime }\right]}^{2} \end{array}$$

## 5 General Case: Local Non‐Coulombic External Potential

Finally, consider the case for which DFT was originally formalized: A system in a local external potential. Equation (5) can be rewritten as

(31) $$\begin{array}{l} \varrho^{F}(\text{r})=4|\nabla {\left(\varrho (\text{r})\right)}^{1/2}{|}^{2} \end{array}$$

That is, the square root of the density and the square root of the Fisher information density satisfy the so‐called eikonal equation:

(32) $$\begin{array}{l} |\nabla {\left(\varrho (\text{r})\right)}^{1/2}|=\frac{1}{2}{\left(\varrho^{F}(\text{r})\right)}^{1/2} \end{array}$$

It can be solved numerically with suitable boundary conditions (see, e.g., [45]). Thus, the density can be in principle obtained from the Fisher information density. As the density determines the external potential, this statement stands for the Fisher information density, too. That is, the first Hohenberg‐Kohn theorem is valid for the Fisher information density. It is proved in the following section that the second Hohenberg‐Kohn theorem also holds for the Fisher information density.

## 6 Variational Principle

According to the second Hohenberg‐Kohn theorem, the ground‐state density $\varrho_{0}$ satisfies the inequality

(33) $$\begin{array}{l} E_{v_{0}}[\varrho_{0}]\leq E_{v_{0}}[\varrho ] \end{array}$$

$E_{v_{0}}$ is the total energy functional of an N‐particle system in the external potential $v_{0}$. ϱ is any density. Equality stands if and only if ϱ equals the ground‐state density.

Define the energy functional of $\varrho^{F}$

(34) $$\begin{array}{l} {\tilde{E}}_{v_{0}}[\varrho^{F}]\equiv E_{v_{0}}[\varrho ] \end{array}$$

That is, the energy $\tilde{E}$ expressed as a functional of the Fisher information density $\varrho^{F}$ is equal to the energy E associated with that density ϱ from which the Fisher information density can be obtained from Equation (5).

From the Equations (33) and (34) we obtain

(35) $$\begin{array}{l} {\tilde{E}}_{v_{0}}[\varrho_{0}^{F}]\leq {\tilde{E}}_{v_{0}}[\varrho^{F}] \end{array}$$

where $\varrho_{0}^{F}$ is the ground‐state Fisher information density, while $\varrho^{F}$ is any Fisher information density. So the variational principle is extended to the Fisher information density.

$\tilde{E}$ can be established by Levy's constrained search. In DFT $E_{v_{0}}$ is obtained by the search for all wave functions providing the given ϱ.

(36) $$\begin{array}{l} E_{v_{0}}[\varrho ]=\min_{\Psi \to \varrho }\langle \Psi |Ĥ|\Psi \rangle \end{array}$$

Here, the search is done for all Ψ yielding $\varrho^{F}$

(37) $$\begin{array}{l} {\tilde{E}}_{v_{0}}[\varrho^{F}]=\min_{\Psi \to \varrho^{F}}\langle \Psi |\hat{H}|\Psi \rangle \end{array}$$

Obviously, any property of the system can be computed if we know Ψ. Therefore, Equations (36) and (37) is in harmony with Equation (34).

## 7 Discussion

Not only is the density a fundamental quantity in chemistry. The density gradient also proved to be important, especially in the quantum theory of atoms in molecules by Bader [46].

In the present theory, the Fisher information density is defined by Equation (5). However, other forms are also possible. An alternative expression for $\varrho^{F}$ by Liu [47] has turned out to be useful in chemical reactivity theory. This expression was compared with the one utilized here Equation (5) for the Neon atom in Reference [47]. Fisher information density was plotted in Reference [48] for the ethylene and benzene molecules.

Nevertheless, the Fisher information density functional theory presented here can only be built on Equation (5).

It is worth mentioning that there are several other descriptors of Coulomb systems [31, 49, 50, 51, 52, 53, 54]. These descriptors are often related to stability, bonding, reactivity, and other physicochemical properties of the system.

In atoms having atomic number Z, there is an upper bound to ϱ(r=0) [55]

(38) $$\begin{array}{l} \varrho (r=0)\leq \frac{2|E|Z}{N\pi }\left(1+\sqrt{1-\frac{2|E|}{NZ^{2}}}\right) \end{array}$$

Equations (30) and (38) lead to an upper bound for an integral of the Fisher information density (providing the density at the nucleus):

(39) $$\begin{array}{l} \frac{1}{4}{\left[\int_{\infty }^{0}(\varrho^{F}{\left((r^{\prime })\right)}^{1/2}dr^{\prime }\right]}^{2}\leq \frac{2|E|Z}{N\pi }\left(1+\sqrt{1-\frac{2|E|}{NZ^{2}}}\right) \end{array}$$

The exact form of the energy functional is unknown at DFT. However, there are a lot of approximations. In the Fisher information density functional theory presented here, the energy is also an unrevealed functional of $\varrho^{F}$. At the moment, it seems rather hard to construct an approximation. Still, the fact that a Fisher information density functional theory can be built provides a novel insight into the relationship between DFT and Fisher information density.

## 8 Conclusion

In DFT, it is often emphasized that the density contains all information about the system. Now, it is shown that an information‐type density, namely, the Fisher information density also grasps all the knowledge of the system. There is a variational principle for the energy as a functional of the Fisher information density. Hohenberg‐Kohn‐like theorems also stand, and the Fisher information density functional theory is now established.

## Conflicts of Interest

The author declares no conflicts of interest.

## Data Availability

Data sharing is not applicable to this article as no datasets were generated or analyzed during the current study.
